# Supplementary material for: Large-scale conformal synthesis of one-dimensional MAX phases
Source: Nat Commun. 2024 Oct 28;15:9275. doi: 10.1038/s41467-024-53137-0 (PMC11519885; doi:10.1038/s41467-024-53137-0)
Supplement: Supplementary file 1 — Supplementary Information of Large-scale conformal synthesis of one-dimensional MAX phases [file 41467_2024_53137_MOESM1_ESM.pdf]

Supplementary Information

Large-scale conformal synthesis of one-dimensional MAX phases

Yuting Li[1,2], Haoran Kong[1,2], Jin Yan[1,2], Qinhuan Wang[1], Xiang Liu[1,2], Mingxue Xiang[1], Yu Wang[1]*

[1]State Key Laboratory of Mesoscience and Engineering, Institute of Process Engineering, Chinese Academy of Sciences, Beijing 100190, P. R. China
[2]School of Chemical Engineering, University of Chinese Academy of Sciences, Beijing 100049, P. R. China
***Corresponding author. Email: wyu@ipe.ac.cn**

Supplementary Text

Synthesis of TiC nanofiber precursor templates

Titanium isopropoxide (TiP) were hydrolyzed in DMF and chloroform solution chelated by acetic acid, therefore the electrospinning solution contained Ti-source (-Ti-O-Ti) and C-source (PVP).

$$Ti(OC_4H_9)_n + x\ H\text{-}OH \rightarrow Ti(OH)(OC_4H_9)_{n-1} + C_4H_9OH \rightarrow Ti(OH)_n + n\ C_4H_9OH \quad (1)$$

$$Ti(OH)_n \rightarrow (\text{-}Ti\text{-}OH + OH\text{-}Ti\text{-})_n \rightarrow \text{-}Ti\text{-}O\text{-}Ti + \text{-}OH \quad (2)$$

Then pristine nanofibers obtained by electrospinning process, and the obtained pristine nanofibers proceeded pre-heating process, where solvents were evaporated and polymeric linear macromolecules of PVP were converted to heat-resistant ladder structures. These ladder structures enable converting more carbon monomers upon following carbonization [1]. When nanofibers heated in vacuum, carbothermal reduction led to the formation of TiC as shown in the following formula.

$$TiO_2 \rightarrow Ti_9O_{17} \rightarrow Ti_6O_{11} \rightarrow Ti_5O_9 \rightarrow Ti_4O_7 \rightarrow Ti_3O_5 \rightarrow Ti_2O_3 \rightarrow Ti(C_XO_y) \rightarrow TiC \quad (3)$$

Since the molar ratio of Ti-source and C-source was larger than 1, there were few carbon residues in nanofiber, even C vacancies could be detected.

Role of TiC_x templates for synthesizing 1D-Ti_2AlC

TiC nanofibers were produced through carbothermal reduction, the carbon source in nanofibers reacted with titanium source and formed TiC crystals along with the amorphous regions. Their long-ranged morphology would not change during the following MS synthesis, hence the synthesized 1D-Ti_2AlC preserved the corresponding morphology.

There have been reported that carbon vacancies in TiC_x enable the rapid intercalation of 'A' sites atoms like Al or Si, thus promoting the formation of MAX phases [2]. Specifically, the migration energy of Al intercalation into TiC was calculated as 0.82 eV, which made Al was easy to incorporate.

In this work, TiC_x was prepared by decreasing the molar ratio of C source in spinning solution and high reduction temperature. The HRTEM results showed that the lattice parameters of 1D-TiC along different direction were larger than standard TiC crystals, suggested that the vacancies substitution on C sites [3]. The Raman spectra also confirmed the existence of vacancies, since the defects would activate the vibrational mode while stoichiometric TiC has a NaCl structure and its crystal symmetry does not have Raman active vibrational modes [3]. Accordingly, the XRD results displayed movement of (111) and (200) peaks shift to larger angle, indicating the increasement of carbon vacancies [4].

It was worth noting that when Ti and Al atoms were added and reacted with 1D-TiC_x, their presence would lead to the formation of more twinning boundaries, stacking faults, and phase boundaries [5,8]. The higher concentration of such defects induced the lower twinning boundary energy (TBE) and stacking faults energy (SFE), which allowed faster and easier diffusion of Ti and Al atoms and increasing the formation rate of Ti_2AlC [2,5].

Roles of molten salts for synthesizing 1D-Ti_2AlC

Molten salts could provide faster mass transfer by convection and diffusion in the liquid phase salts, thus redressing the slow diffusion limitations in solid-phase reactions. Moreover, molten salt is ionic liquid at high temperatures, therefore facilitates chemical reactions by introducing a strong polarizing force that destabilize metallic, ionic, or covalent bonds [6]. Eutectic NaCl/KCl used in this work has a low melting point 658 °C [7], which was much lower than reaction temperature. Although it is still unclear to understand the reaction details in molecular level in ionic molten salt, there have been researched that metal atoms are more easily dissolved in molten salt than in solid state reactions by theoretical calculations [8].

In this work, Ti and Al as added necessary atoms in molten salts participated reactions in their ionic forms. Al metal has low melting point around 660 °C, while Ti metal has higher melting point. However, it was shown that Ti can be dissolved partially in chloride salts by forming Ti^{2+} and Ti^{3+} through disproportionation reactions [9]. Then Ti atoms were continually transferred into ions and reacted with Al to produce TiAl intermetallic compounds, and finally Ti_2AlC were synthesized at relatively low temperature.

XPS analysis

The surface atomic bonding state of synthesized 1D-Ti_2AlC samples was characterized by XPS analysis. The Ti 2p region could be deconvoluted into the bonding of Ti–C and Ti–O respectively, matching the $2p_{3/2}$ peaks at binding energies of 455.04 and 458.95 eV, respectively, with their respective spin-orbit splitting $2p_{1/2}$ peaks that differing about 6 eV, where at 460.99, and 464.65 eV. The Al 2p spectra shows Al–Ti and Al–O peaks at around 72.2 and 74.8 eV, respectively. The Al-O fraction is predominant over Al-Ti fraction, indicating that aluminum exhibits sensitivity to oxygen at the surface of MAX phases. The binding energies of C 1*s* at 281.3, 284.6, 286 and 288.6 eV represented C–Ti, C–C, C–O, and C=O, respectively [10-12]. The ratio of the corresponding fractions was summarized in Supplementary Table 4.

Synthesis parameters control of 1D-Ti_2AlC

The reaction parameters for molten salts synthesis were analyzed. Firstly, the content of mixture powder of Ti, Al and salts was controlled. The XRD spectra illustrated that small amount of powder mixture would decrease the yield of Ti_2AlC. Since the powder mixture was covered onto the TiC nanofiber membranes instead of blending with each other completely by griding, Ti, Al atoms in a lower amount of mixture powder would be insufficient for Ti_2AlC synthesizing, i.e., the molar ratio of Ti, Al and TiC that contacting was less than 1: 1: 1. Contrarily, higher amount of mixture powder offered excessive reactants for complete transformation of Ti_2AlC, although there were intermetallic compound residues adhered on the nanofibers, which could be easily washed by HCl. The influence of molar ratio of metal atoms and salts in mixture powder was also analyzed. Higher content of salts could provide more suitable environment for reaction and retain the morphology better, but lower content of salts also met the reaction requirement once Ti and Al atoms were excessive. To investigate the most appropriate reaction duration, the XRD spectra with different reaction time were obtained. When

reaction time was 10 mins, the major phase was TiC and TiAl since the reaction was only triggered in a small part of nanofibers. With increased reaction time, the major phase was changing into Ti_2AlC, and the amount of TiC decreased consequentially with complete crystalline reconstruction.

Synthesis of 1D-Ti_3AlC_2

The synthesized 1D-Ti_3AlC_2 owned high purity and long-ranged nanofibrous morphology. The *c* parameter was consistent with XRD refinement results, and its alternating stacking sequence was clearly observed through HRTEM results. The electron energy-loss spectrometer (EELS) confirmed the atomic layer was composed of Ti, Al and C.

XRD pattern of 1D-Ti_3AlC_2 from 1000 °C reaction for 0.5 hours without HCl washing indicated that the byproducts were TiC and Al_2O_3, since the added atoms were only Al atoms.

The TG-DSC curves revealed a similar formation process compared with 1D-Ti_2AlC. The sharp, small endothermic peak emerges around 650 °C, indicating the melt of the salt mixture and Al atoms. Since there were no Ti atoms in mixture powder, thus the next stage indicated the diffusion of melting Al into the ordered carbon vacancies [2]. The evident exothermic peak observed at 926 °C represented the formation of Ti_3AlC_2 through the diffusion between TiC_x and Al, which was higher than the formation temperature of Ti_2AlC. The sharp exothermic peak at 1010 °C illustrated the vaporization of molten salts, and the caused mass loss led to the absence of NaCl and KCl peaks in the XRD results.

The HRTEM results also revealed the in-situ evolution during the synthesis of 1D-Ti_3AlC_2, the disordered $(TiAl)C_x$ and ordering Ti_3AlC_2 atomic arrangements were observed.

Synthesis of 1D-Ti_3SiC_2

The synthesized 1D-Ti_3SiC_2 owned high purity and long-ranged nanofibrous morphology. The *c* parameter was consistent with XRD refinement results, and its alternating stacking sequence was observed through HRTEM results.

XRD pattern of 1D-Ti_3SiC_2 from 1100 °C reaction for 0.5 hours without HCl washing indicated that the byproducts were Ti_5Si_3, $TiSi_2$, and unreacted TiC. The Ti-Si intermetallic compounds would react with TiC_x to form 1D-Ti_3SiC_2 further [13].

The TG-DSC curves revealed a similar formation process. The sharp, small endothermic peak emerges around 650 °C, indicating the melt of the salt mixture and Al atoms. The third stage represents the formation of Ti-Si intermetallic compounds. The evident exothermic peak at 961 °C in the next stage represented the formation of Ti_3SiC_2. The sharp exothermic peak at 1006°C illustrated the vaporization of molten salts.

The HRTEM results also revealed the in-situ evolution during the synthesis of 1D-Ti_3SiC_2, the disordered $(TiSi)C_x$ and ordering Ti_3SiC_2 atomic arrangements were observed.

Synthesis of 1D-Ti_2SnC

The synthesized 1D-Ti_3SiC_2 owned high purity and long-ranged nanofibrous morphology. The *c* parameter was consistent with XRD refinement results, and its alternating stacking sequence was observed through HRTEM results.

XRD pattern of 1D-Ti_3SiC_2 from 850 °C reaction for 0.5 hours without HCl washing indicated that the byproducts were Ti_2Sn, and Ti_3Sn. The Ti-Sn intermetallic compounds would react with TiC_x to form 1D-Ti_3SiC_2 further [14].

The TG-DSC curves revealed a similar formation process. A sharp, small endothermic peak emerges around 653 ℃, indicating the melt of the salt mixture and Al, Sn atoms. The third stage represents the formation of Ti-Si intermetallic compounds. The evident exothermic peak at 834 ℃ in the next stage represented the formation of Ti_2SnC. The sharp exothermic peak at 1054 ℃ illustrated the vaporization of molten salts.

The HRTEM results also revealed the in-situ evolution during the synthesis of 1D-Ti_2SnC, the disordered $(TiSn)C_x$ and ordering Ti_2SnC atomic arrangements were observed.

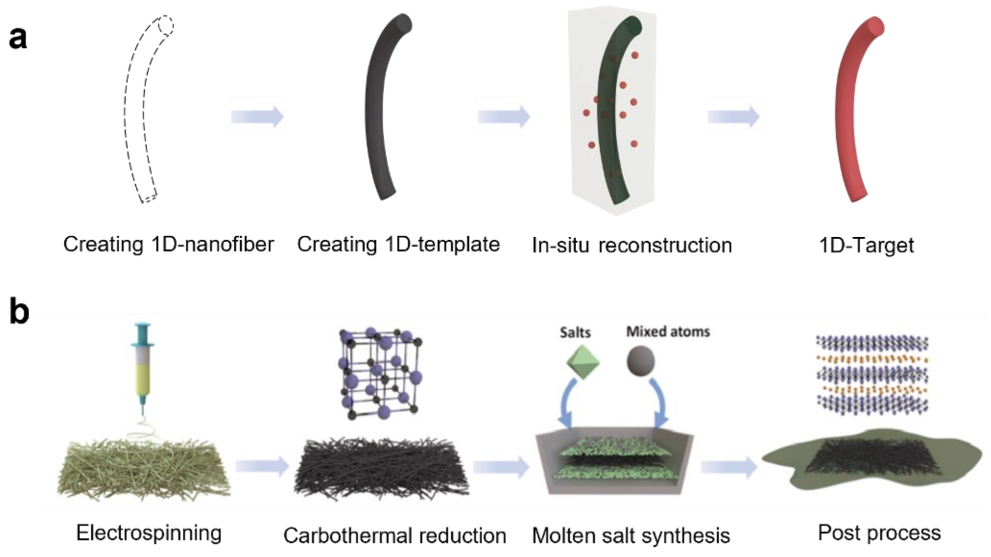

Supplementary Figure 1: Schematic illustration of the conformal strategy and synthetic protocol for Ti_2AlC nanofibers. (a) Steps of conformal strategy for synthesizing 1D-MAX phases. (b) Steps in this work for synthesizing 1D-Ti_2AlC. TiC nanofibers as templates were firstly obtained by electrospinning and carbothermal reduction. The mixed atoms including Ti and Al atoms in this case then added along with eutectic molten salts to cover the TiC nanofiber. 1D-Ti_2AlC then formed through in-situ crystalline evolution and reconstruction in molten salts.

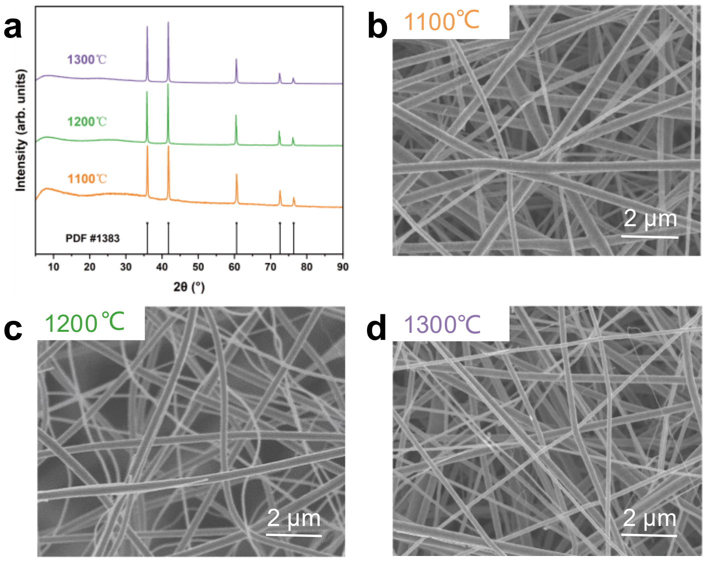

Supplementary Figure 2: Characterization of TiC nanofibers with different carbothermal reduction temperatures. (a) XRD patterns show higher crystallinity with higher temperature. (b), (c), and (d) are the corresponding SEM images, which illustrate the decreasing diameters with higher temperatures. By considering both the flexibility and crystallinity, TiC nanofibers obtained from 1200 °C were chosen as the template in this work unless stated otherwise.

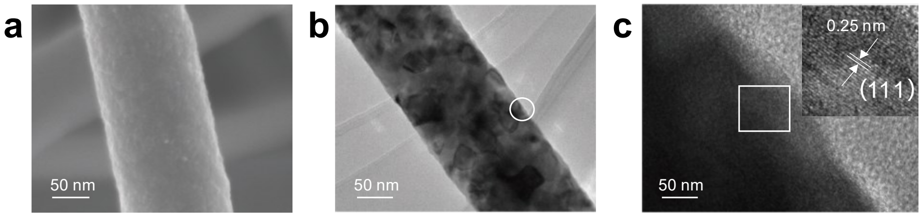

Supplementary Figure 3: SEM image (a), and TEM image (b) confirmed that the diameter of TiC nanofiber was about 150 nm. TEM image (b) represents that TiC nanofiber was constructed by TiC nano crystals and amorphous areas, which provided efficient defects for migration and diffusion of added A sites atoms to facilitate the formation of 1D-MAX phases. TEM image (c) is the enlargements in (b), with insert show the TiC nanocrystal structure.

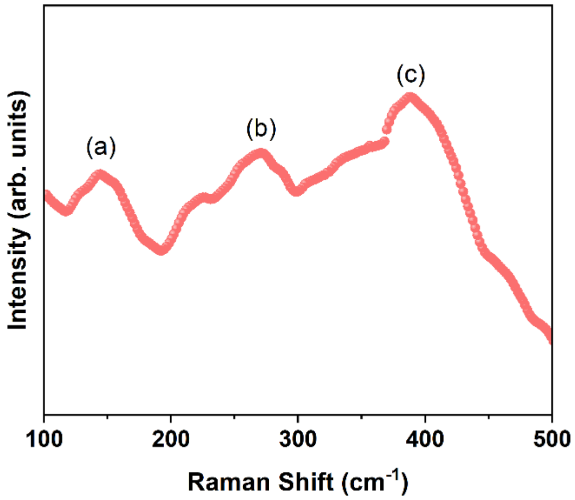

Supplementary Figure 4: Raman spectra of Ti_2AlC nanofiber. The corresponding peaks from (a) to (c) represented the ω_1 (144 cm^{-1}), ω_2 & ω_3 (223 and 269 cm^{-1}) and ω_4 (388 cm^{-1}). The calculated Raman peaks of Ti_2AlC are 149, 248 & 262, and 387 cm^{-1} accordingly[69].

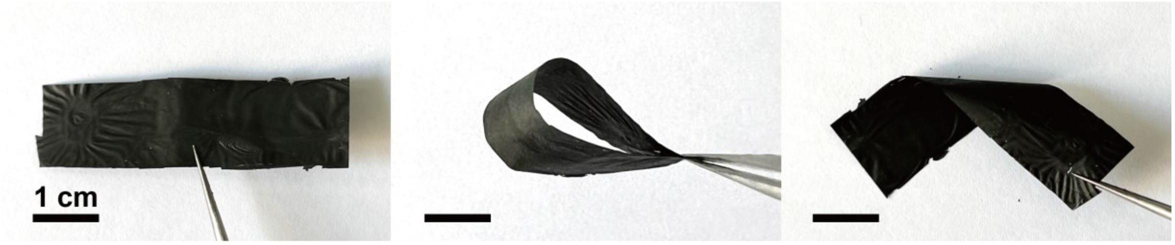

Supplementary Figure 5: Photograph of Ti_2AlC nanofiber membrane presented the excellent free-standing ability and flexibility.

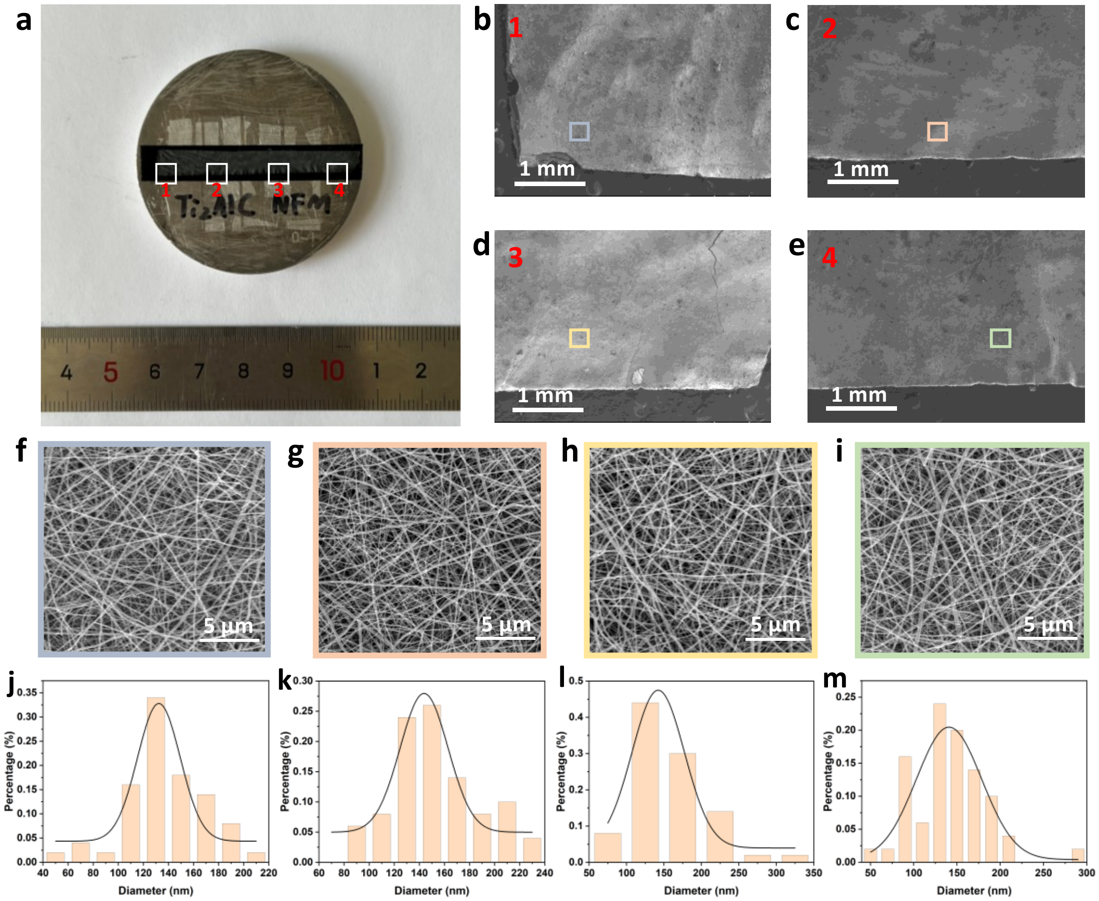

Supplementary Figure 6: SEM images for measuring the average diameters of 1D-Ti_2AlC. (a) A long piece of Ti_2AlC nanofiber membrane attached on the SEM stage. The selected areas are enlarged in (b) to (e), and the corresponding surface morphologies are presented from (f) to (i). The statics of (j) to (m) are analyzed from (f) to (i), respectively.

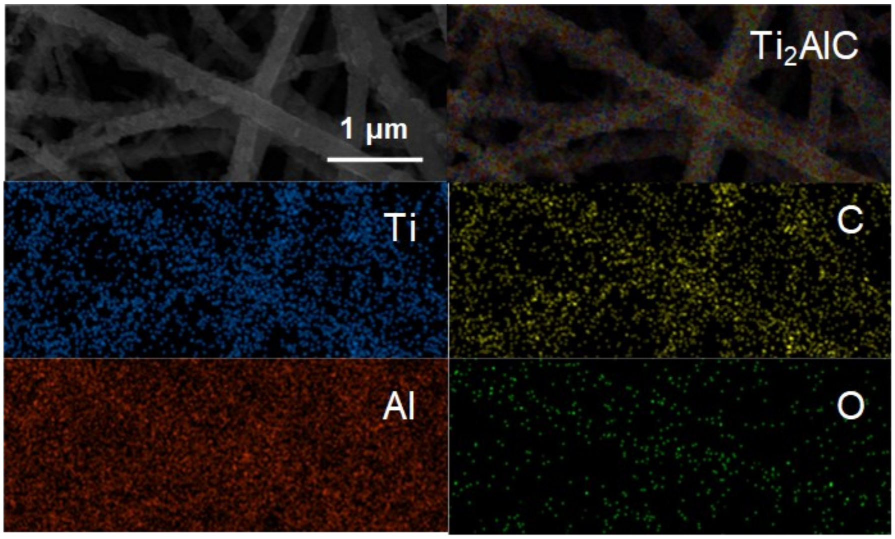

Supplementary Figure 7: SEM-EDS mapping of all elements of 1D-Ti_2AlC.

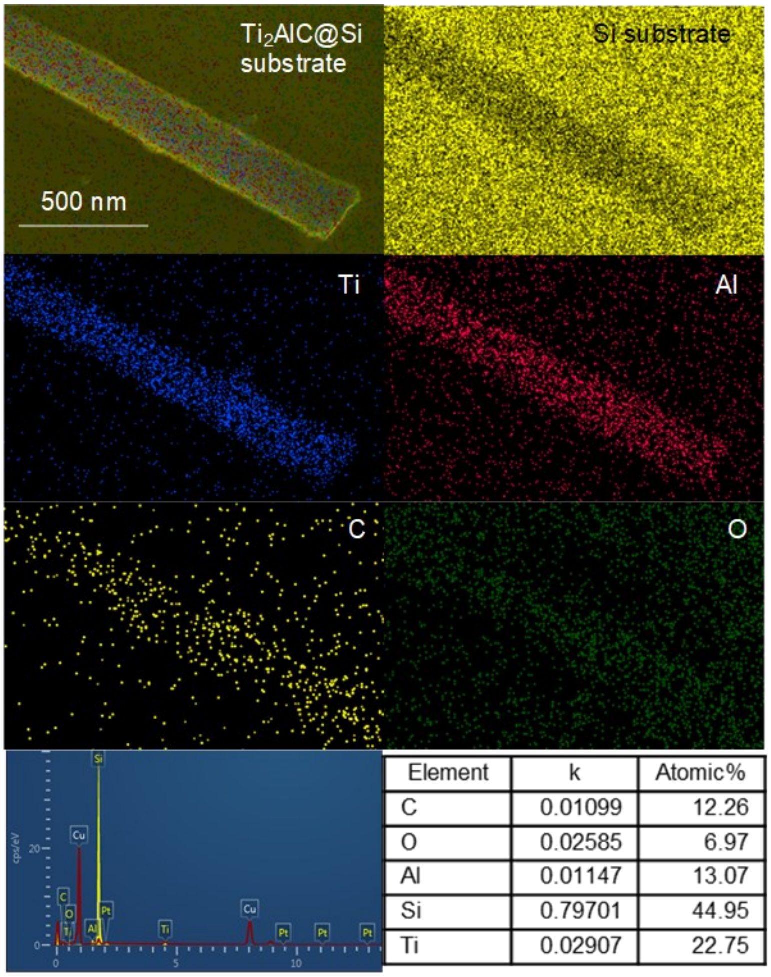

Element	k	Atomic%
C	0.01099	12.26
O	0.02585	6.97
Al	0.01147	13.07
Si	0.79701	44.95
Ti	0.02907	22.75

Supplementary Figure 8: SEM-EDS mapping of all elements of 1D-Ti_2AlC for samples at Si substrate. The distribution of major elements are clearly shown across the nanofiber, and the ratio is around 2 : 1 : 1, which is corresponding to the stoichiometry of Ti_2AlC. Noting that the yellow spectrum is the actual spectrum for Si substrate and the red one is for comparison with the spectrum in figure S9.

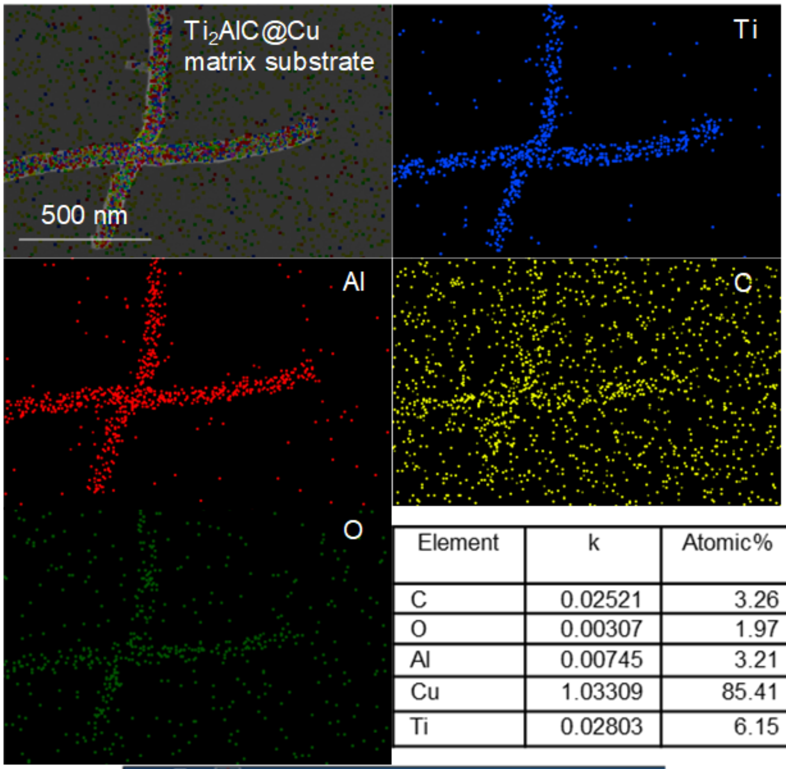

Element	k	Atomic%
C	0.02521	3.26
O	0.00307	1.97
Al	0.00745	3.21
Cu	1.03309	85.41
Ti	0.02803	6.15

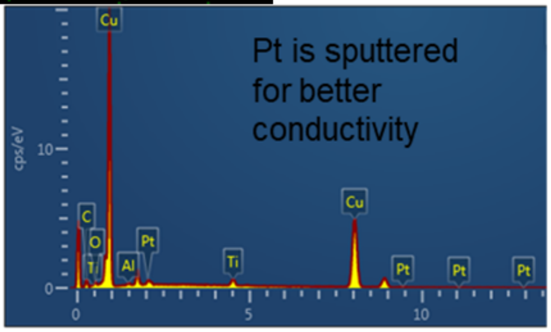

Supplementary Figure 9: SEM-EDS mapping of all elements of 1D-Ti_2AlC for samples at Cu matrix substrate. The distribution of major elements are clearly shown across the nanofiber, and the ratio is around 2 : 1 : 1, which is corresponding to the stoichiometry of Ti_2AlC. It is worth noting that the carbon element from the carbon cloth in the copper matrix substrate and the oxygen element from the silicon wafer may be detected by EDS, which means that the quantitative analysis ratios obtained from figure S8 and figure S9 may not be entirely accurate and should be considered as reference data only.

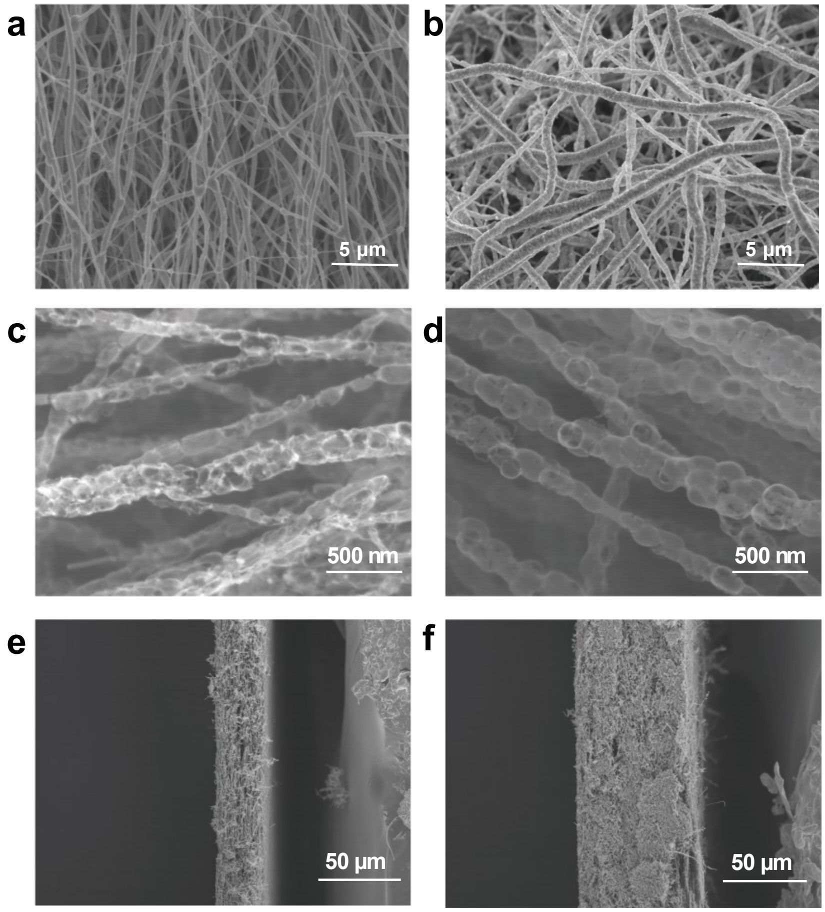

Supplementary Figure 10: Morphologies of 1D-Ti_2AlC with various TiC nanofiber templates by designed electrospinning process. (a) General nanofibers with thinner diameters. (b) Nanofibers with larger diameters by decreasing electrospinning voltages. (c) and (d) were porous nanofibers by replacing PVP into PTFE. (e) Nanofiber membranes with small thickness while (f) larger thickness were obtained by prolonging spinning time.

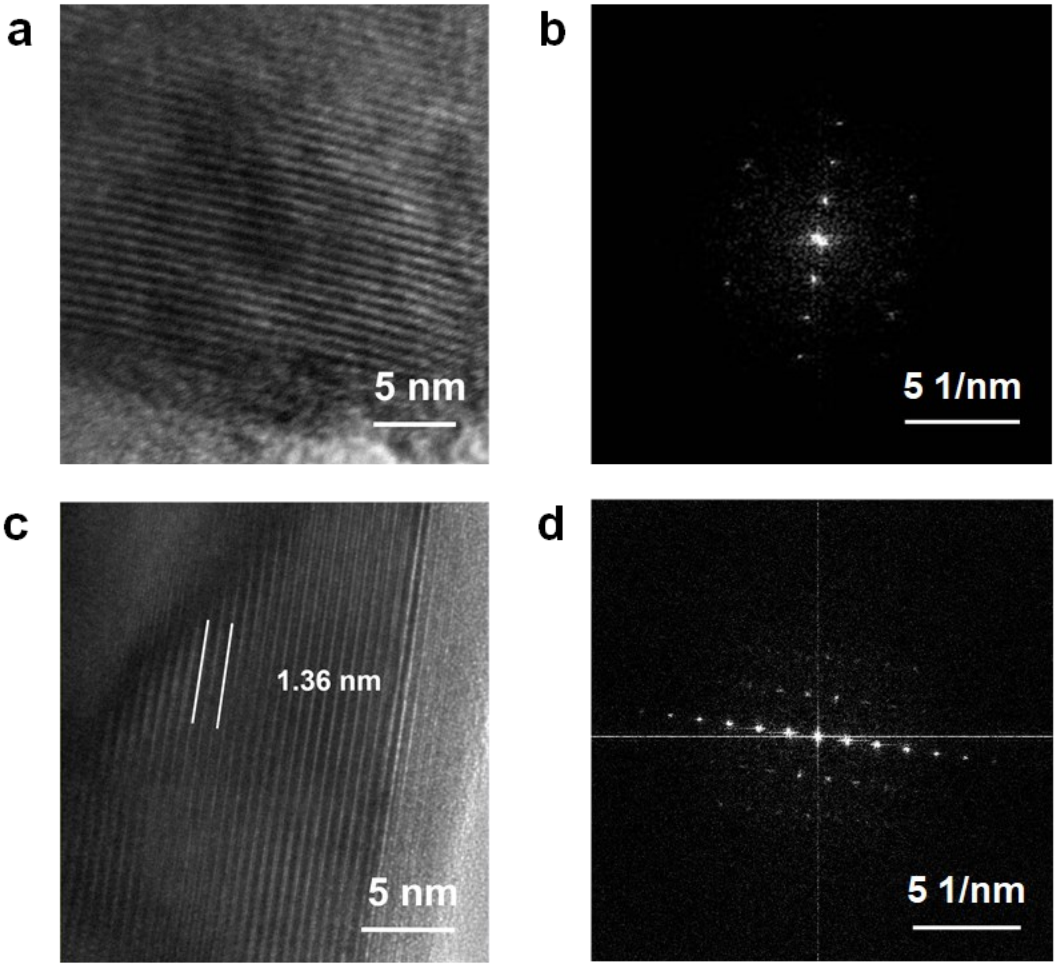

Supplementary Figure 11: (a) TEM image of 1D-Ti_2AlC from figure 1f in manuscript, and (b) is its corresponding FFT pattern; (c) TEM image of 1D-Ti_2AlC from figure 1g in manuscript, and (d) is its corresponding FFT pattern.

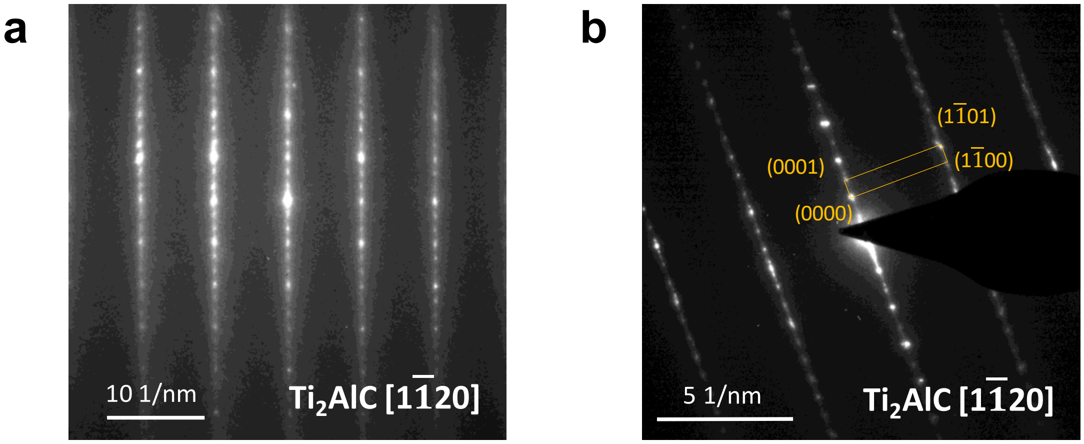

Supplementary Figure 12: SAED patterns of 1D-Ti_2AlC with incident beam along the [1$\bar{1}$20] direction.

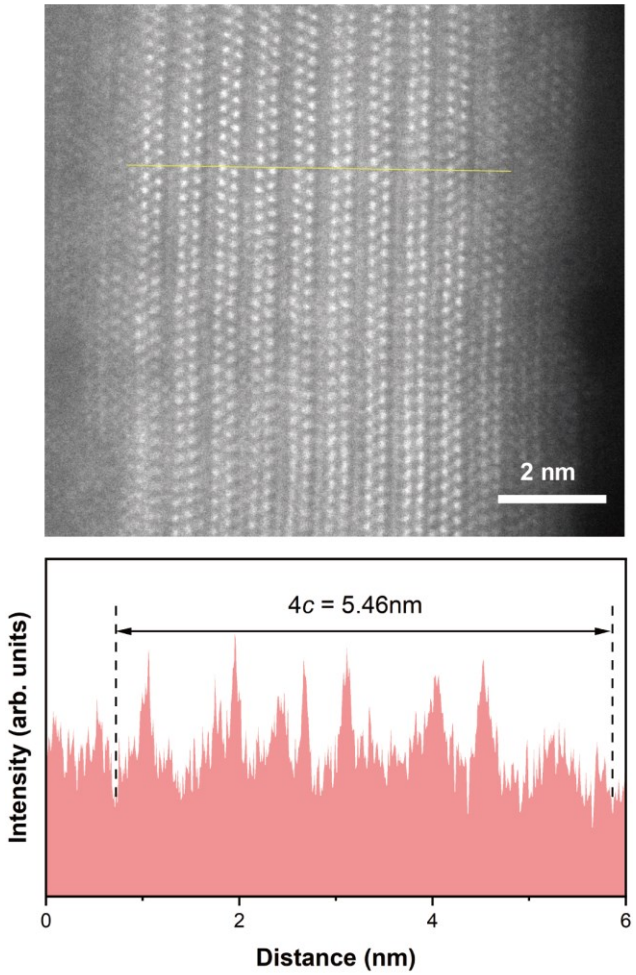

Supplementary Figure 13: Atomic structural analysis of the *c* lattice parameter of 1D-Ti_2AlC. STEM
image and corresponding HAADF intensity profile across the layers along the $[11\bar{2}0]$ axis

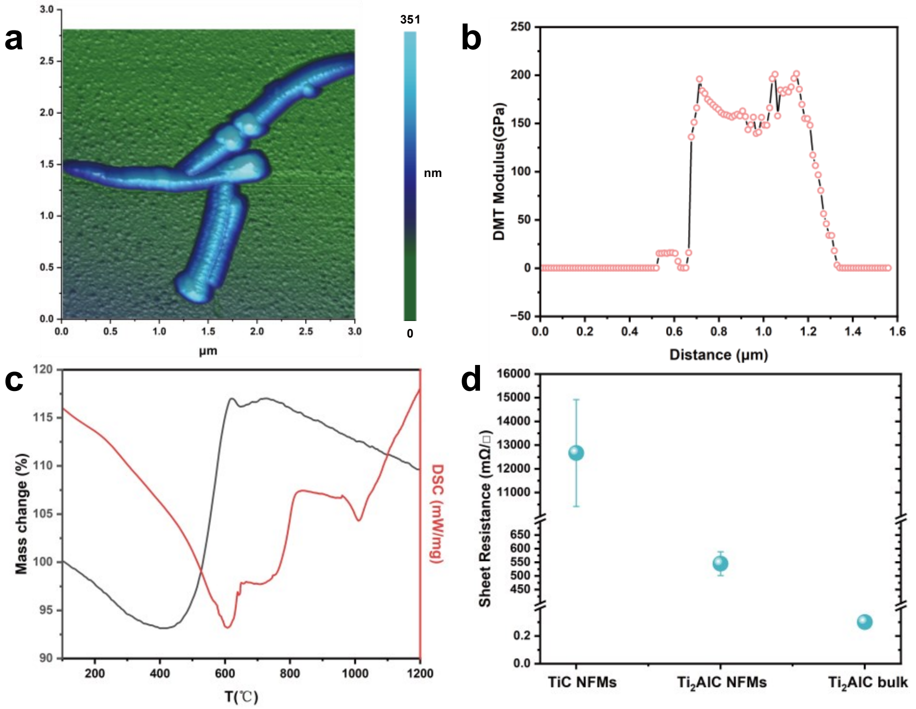

Supplementary Figure 14: Properties of 1D-Ti_2AlC. (a) Atomic force microscope (AFM) topography image of Ti_2AlC nanofibers. (b) The distribution of elastic modulus along the location marked by the yellow line in (a). (c) TG-DSC curves recorded during heating of Ti_2AlC nanofibers up to 1000 °C at a rate of 10 °C/min in flowing air. (d) Sheet resistances of TiC nanofiber membranes, Ti_2AlC nanofiber membranes, and Ti_2AlC bulks, where the error bars represent the standard error of the mean resistance for each group, based on n = 3 independent experiments.

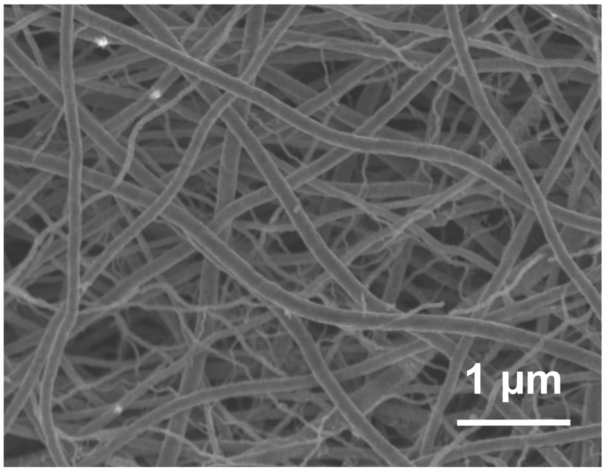

Supplementary Figure 15: The morphology of assembled nanofiber membranes was constituted of 1D-Ti_2AlC building blocks with smooth surface and uniform diameters.

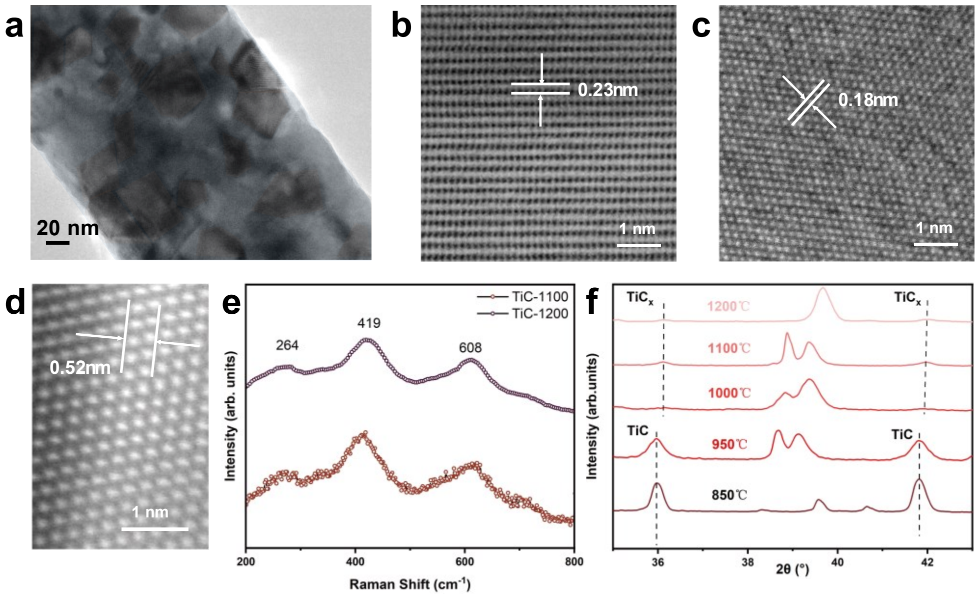

Supplementary Figure 16: Characterizations of TiC_x nanofibers. (a) TEM image of crystalline and amorphous regions of TiC_x nanofiber. HRTEM images of TiC_x at (b) [010], (c) [220] and (d) [-101] zone axis orientation. The lattice parameters were larger than TiC (PDF#32-1383), suggested the vacancies of carbon atoms. The Raman spectrum (e) of TiC nanofibers synthesis at 1100 ℃ and 1200 ℃ were matched with other reports, and the corresponding vibration modes were not appeared if the crystal were defects-less TiC. The XRD spectrum (f) from 850 ℃ to 1200 ℃ of Ti_2AlC synthesis also indicated the peak shifts to higher angle, suggested the vacancies replacements of C atoms and the consequential TiC_x formation.

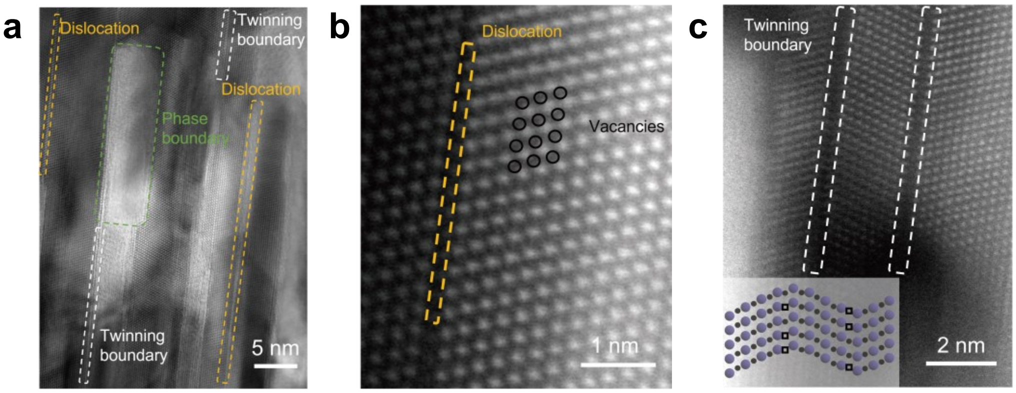

Supplementary Figure 17: The defects in TiC_x nanofibers were observed through HRTEM of (a) line defects, (b) point defects and (c) magnification of twinning boundaries in (a).

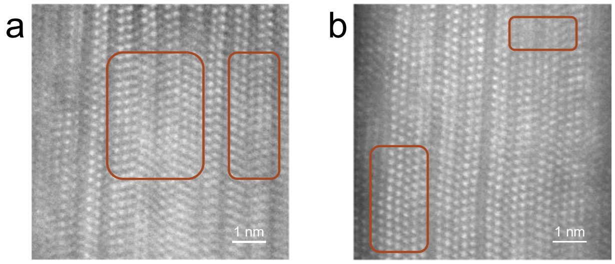

Supplementary Figure 18: The observation of $(TiAl)C_x$ and disordered Ti_2AlC in red frames by HRTEM images, where (a) and (b) were selected areas from 1D-Ti_2AlC from 900 °C reaction with dwell time 1 hour.

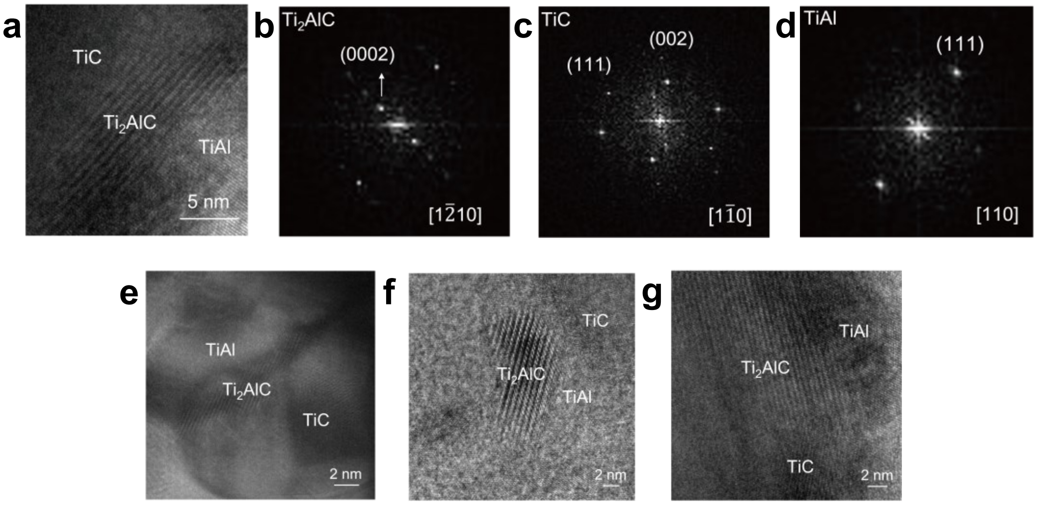

Supplementary Figure 19: TEM images of products with different reaction temperatures revealed the synthesis mechanisms in atom-level. (a) TEM results at 900 ℃ show the grains of Ti_2AlC, TiC and TiAl and their corresponding FFT images (b), (c) and (d). TEM images of products with other reaction temperatures were shown in (e) 850 ℃, (f) 950 ℃, and (g) 1000 ℃ and owned same components, suggested that Ti_2AlC were nucleated through diffusion of TiC_x and TiAl.

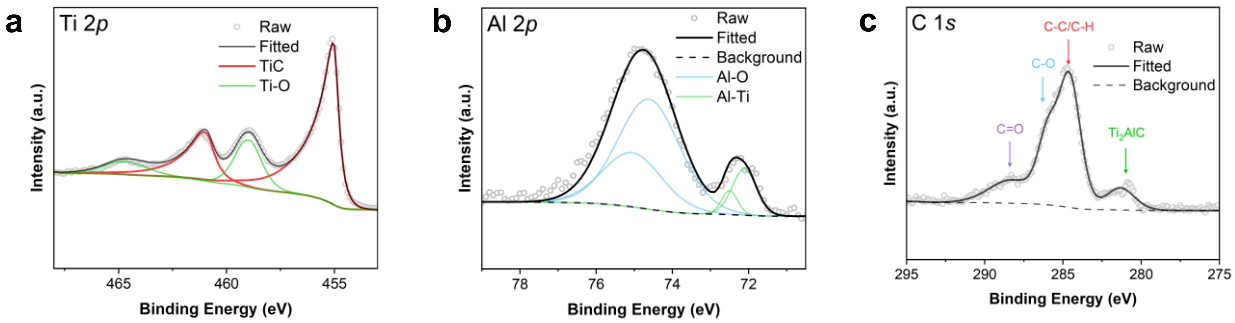

Supplementary Figure 20: Deconvolution of (a) Ti 2p, (b) Al 2p, and (c) C 1s XPS spectra for 1D-Ti_2AlC samples at reaction temperature of 900 °C.

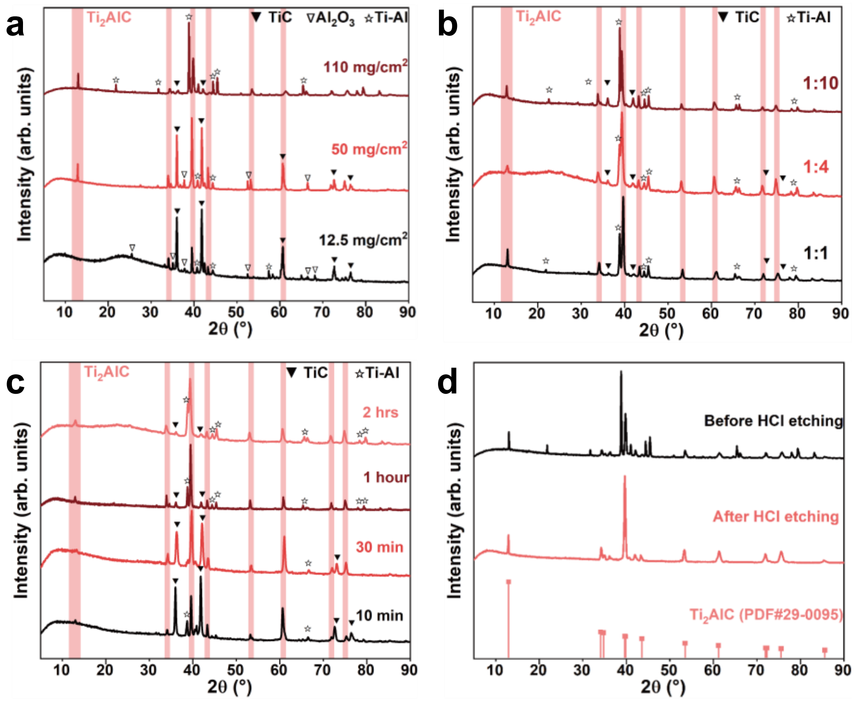

Supplementary Figure 21: XRD patterns with different reaction parameters for synthesizing Ti$_2$AlC nanofibers. (a) Contents of molten salts. (b) Molar ratio of adding atoms and salts. (c) Reaction dwell time at 900 °C syntheses. (d) With and without washing by HCl solution.

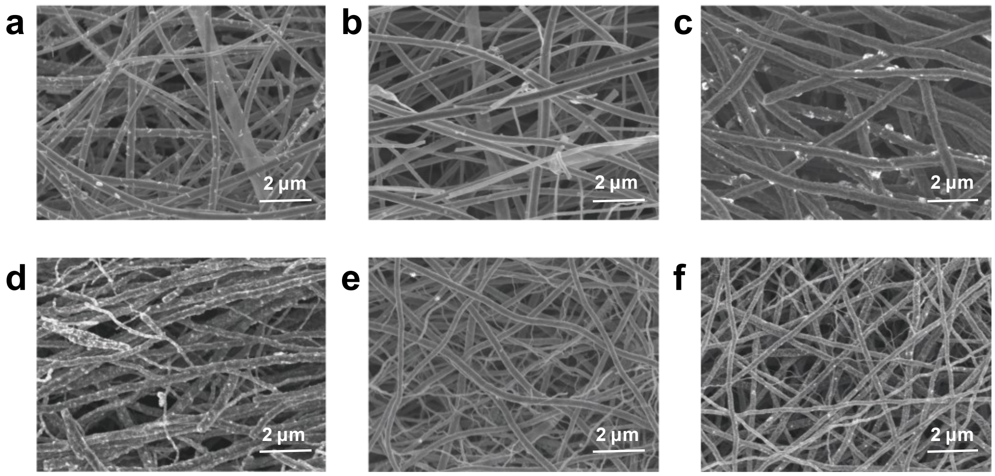

Supplementary Figure 22: SEM images demonstrated the retention of nanofiber morphology of 1D-Ti_2AlC at different reaction temperatures (a) 800, (b) 850, (c) 900, (d) 1000, (e)1100, and (f) 1200 °C.

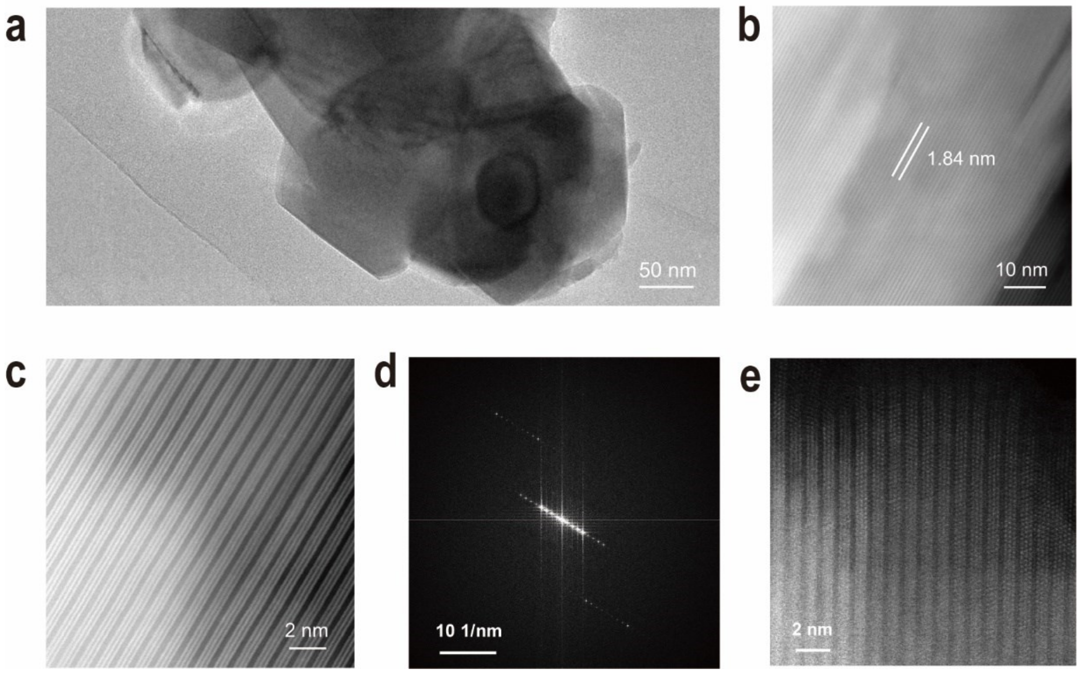

Supplementary Figure 23: TEM results of 1D-Ti_3AlC_2. (a) Nanofibrous morphology with stacking crystals of Ti_3AlC_2. (b) The *c* parameter of 1D-Ti_3AlC_2 was measured and consistent with XRD results. (c) HRTEM image showed stacking sequences of $Ti_3AlC_2$with three Ti layers and one Al layer. (d) The corresponding FFT pattern of (c). (e) The HRTEM image from another area of 1D-Ti_3AlC_2.

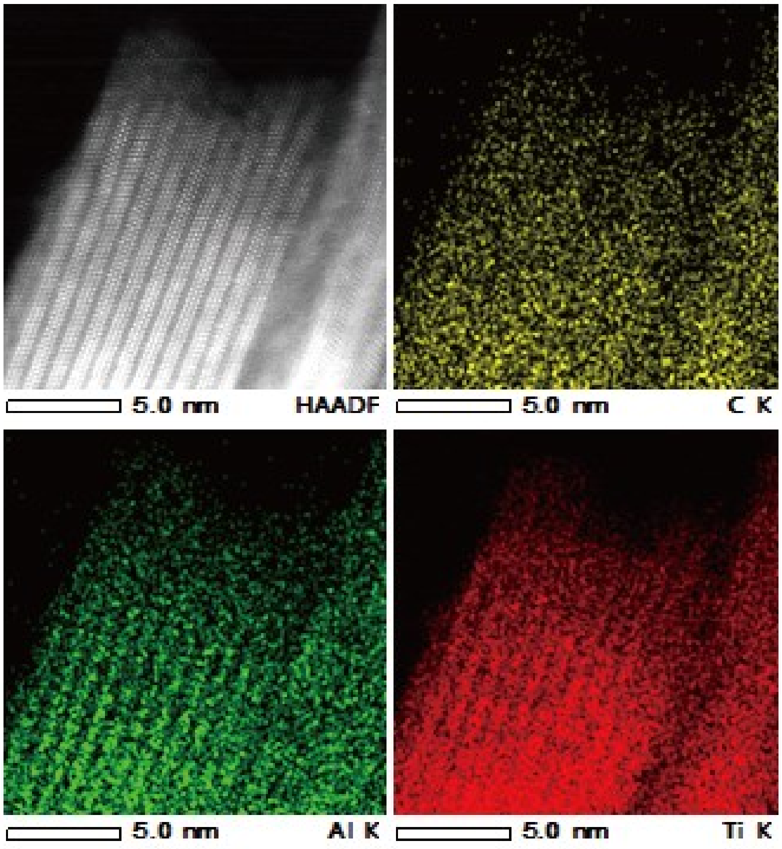

Supplementary Figure 24: HAADF and EESL elemental mapping of 1D-Ti_3AlC_2 along the $[11\bar{2}0]$ axis.

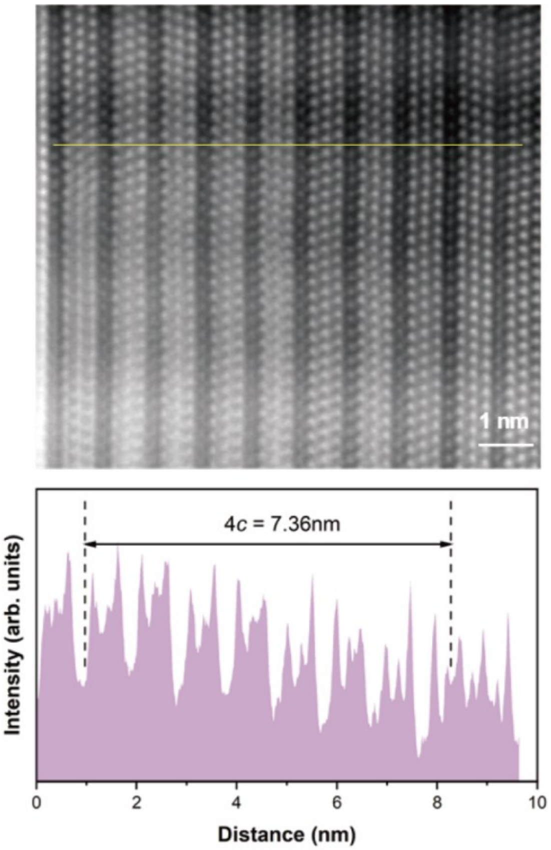

Supplementary Figure 25: Atomic structural analysis of the *c* lattice parameter of 1D-Ti_3AlC_2. STEM
image and corresponding HAADF intensity profile across the layers along the $[11\bar{2}0]$ axis.

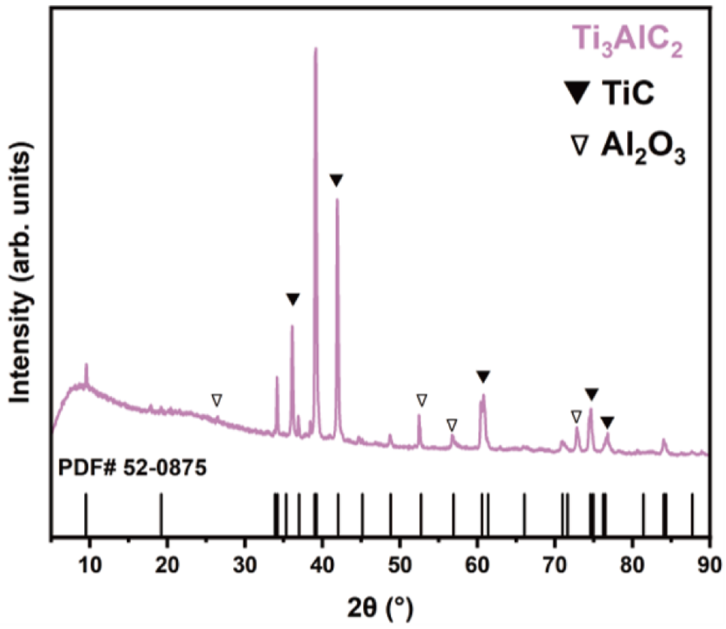

Supplementary Figure 26: XRD pattern of 1D-Ti_3AlC_2 from 1000 °C reaction for 0.5 hours without HCl washing. The products conclude major phase Ti_3AlC_2, and a small amount of TiC and Al_2O_3.

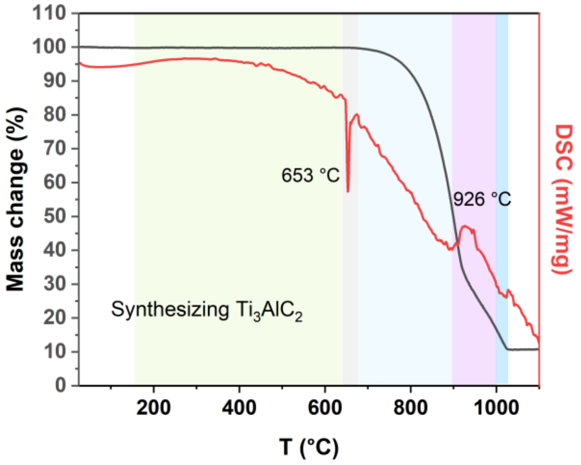

Supplementary Figure 27: TG-DSC curve for synthesizing 1D-Ti_3AlC_2 from room temperature to 1100 ℃ in Ar atmosphere. The colored regions presented melting of eutectic salts and Al metal atoms (653 ℃), formation of intermetallic compounds, formation of Ti_3AlC_2 (926 ℃), and evaporation of salts (1010 ℃).

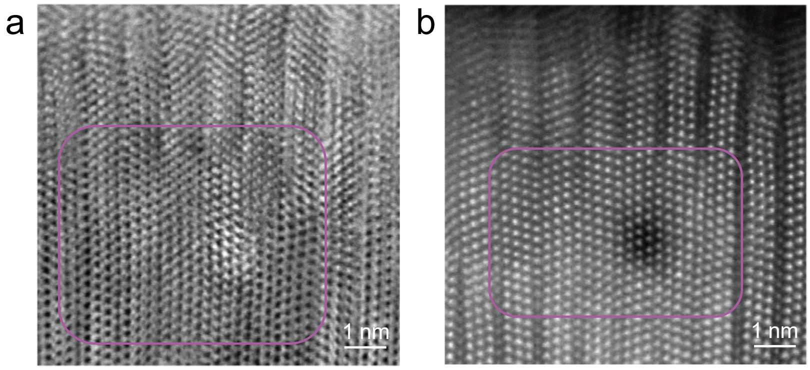

Supplementary Figure 28: The observation of $(TiAl)C_x$ and disordered Ti_3AlC_2 in pink frames by HRTEM images, where (a) and (b) were selected areas from 1D-Ti_3AlC_2 from 1000 ℃ reaction with dwell time 0.5 hours.

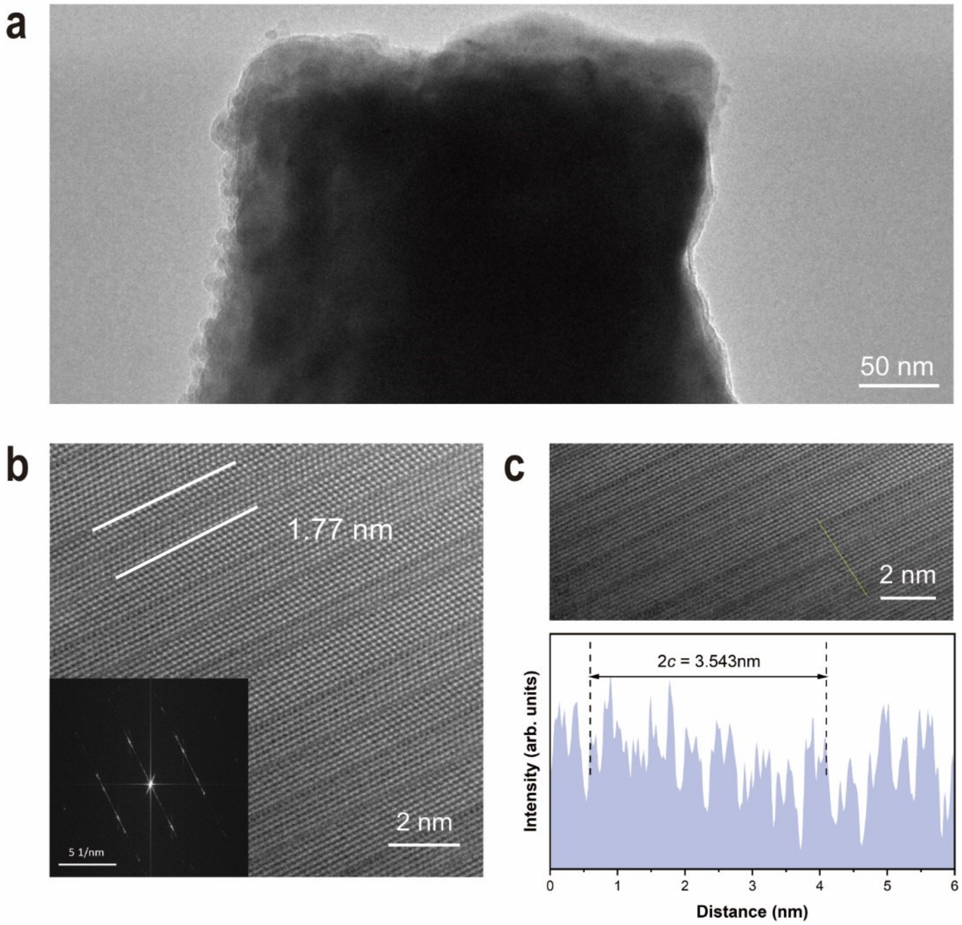

Supplementary Figure 29: TEM results of 1D-Ti_3SiC_2. (a) Nanofibrous morphology of 1D-Ti_3AlC_2. (b) The *c* parameter of 1D-Ti_3SiC_2 was measured and consistent with XRD results, the insert was the corresponding FFT pattern. (c) HRTEM image showed stacking sequences of Ti_3SiC_2 and corresponding HAADF intensity profile across the layers along the [11$\bar{2}$0] axis.

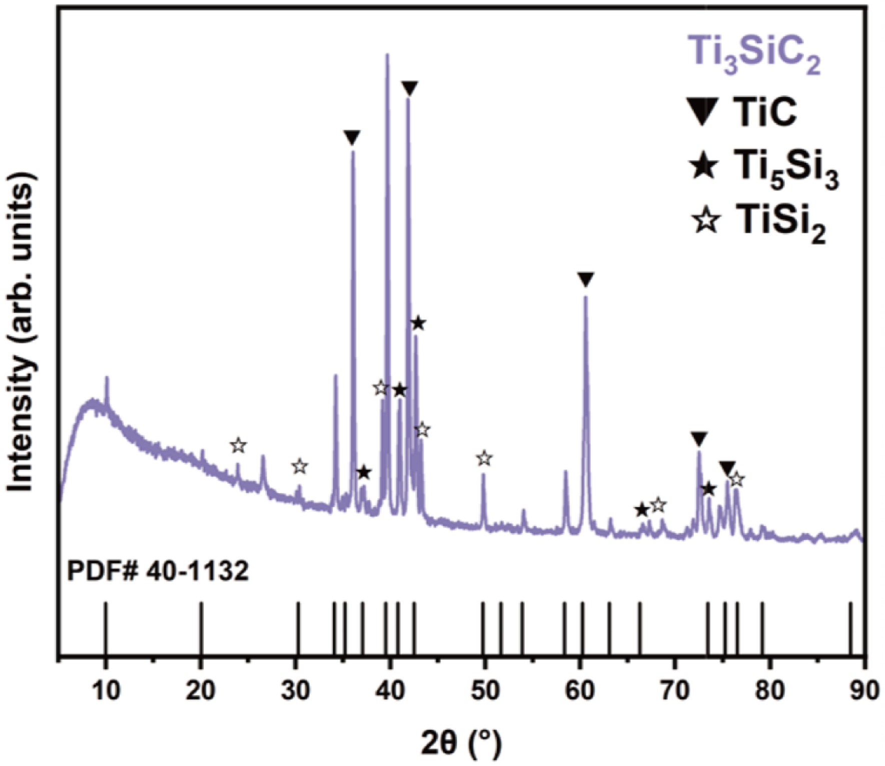

Supplementary Figure 30: XRD pattern of 1D-Ti_3SiC_2 from 1100 °C reaction for 0.5 hours without HCl washing. The products conclude major phase Ti_3SiC_2, and a small amount of TiC, Ti_5Si_3, and $TiSi_2$, which were the common intermetallic compounds during synthesizing Ti_3SiC_2.

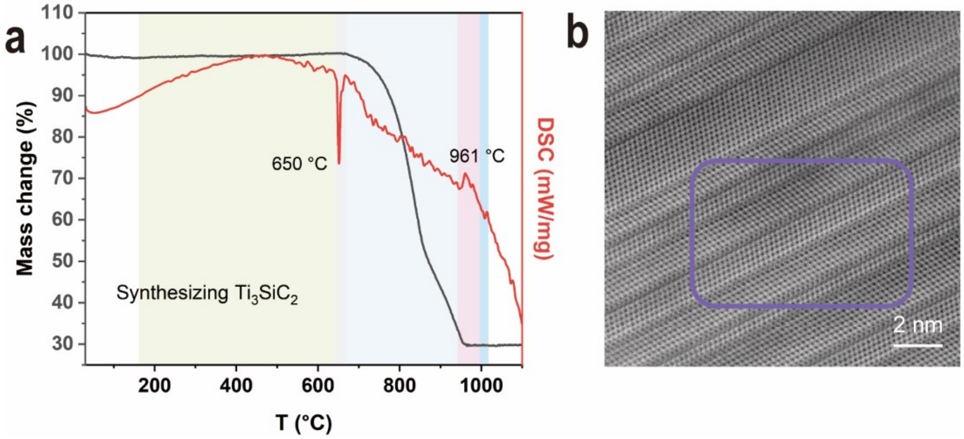

Supplementary Figure 31: (a) TG-DSC curve for synthesizing 1D-Ti_3SiC_2 from room temperature to 1200 °C in Ar atmosphere. The divided regions presented solid solution formation of salts, melting of eutectic salts (650 ℃), formation of intermetallic compounds, formation of Ti_3SiC_2 (961 ℃), and evaporation of salts (1006 ℃). (b) The observation of $(TiSi)C_x$ and disordered Ti_3SiC_2 in violet frames by HRTEM images from selected areas of 1D-Ti_3SiC_2 at 1100 ℃ reaction with dwell time 0.5 hours.

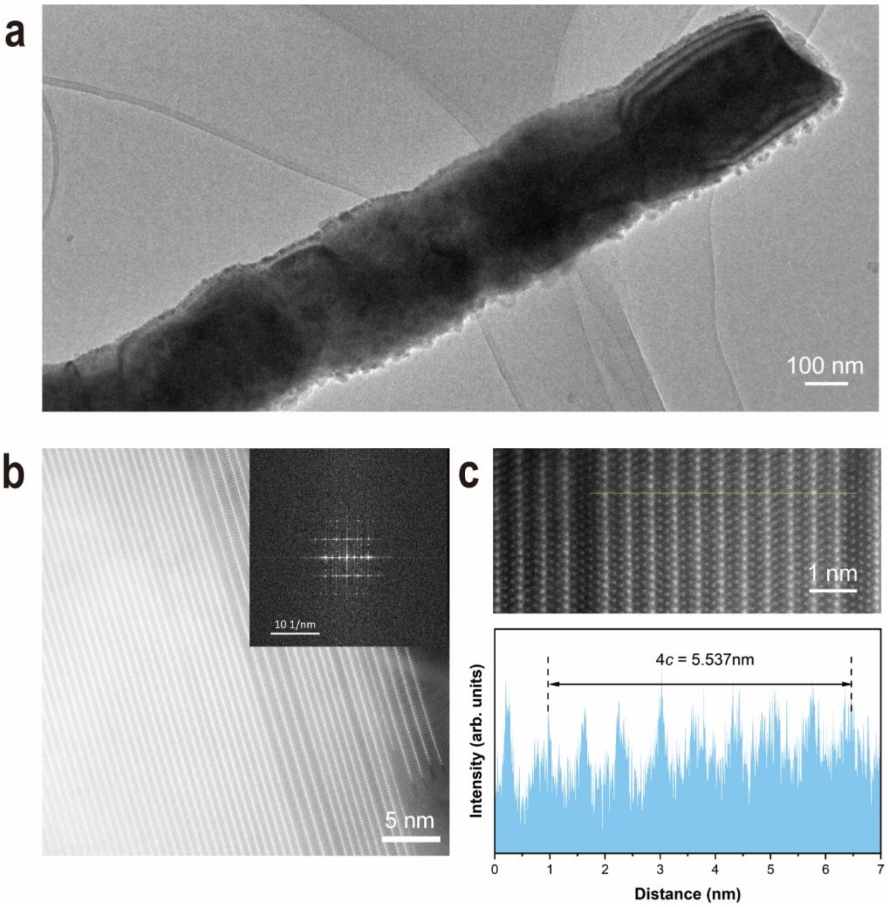

Supplementary Figure 32: TEM results of 1D-Ti_2SnC. (a) Nanofibrous morphology of 1D-Ti_2SnC. (b) The typical layered structure of Ti_2SinC, with the corresponding inserting FFT pattern. (c) HRTEM image measured *c* parameter of 1D-Ti_2SnC and consistent with XRD results from the HAADF intensity profile across the layers along the $[11\bar{2}0]$ axis.

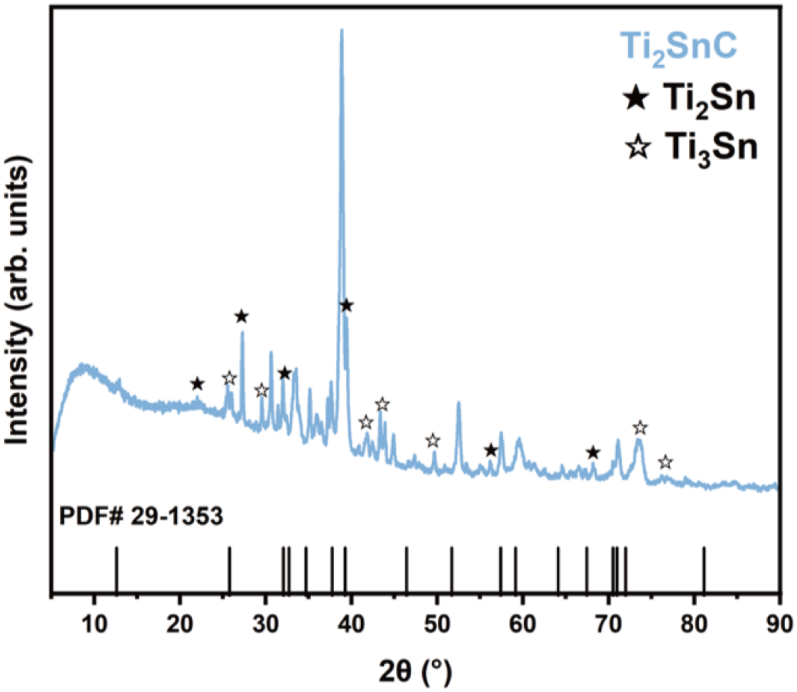

Supplementary Figure 33: XRD pattern of 1D-Ti_2SnC from 850 °C reaction for 0.5 hours without HCl washing. The products conclude major phase Ti_2SnC, and a small amount of Ti_2Sn, and Ti_3Sn, which were the common intermetallic compounds during synthesizing Ti_2SnC.

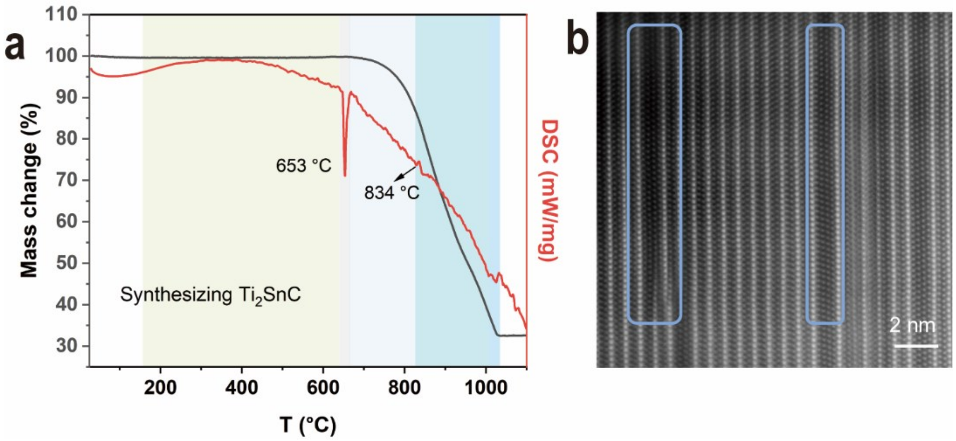

Supplementary Figure 34: (a) TG-DSC curve for synthesizing 1D-Ti_2SnC from room temperature to 1200 °C in Ar atmosphere. The divided regions presented solid solution formation of salts, melting of eutectic salts (653 °C), formation of intermetallic compounds, formation of Ti_2SnC (834 °C), and evaporation of salts (1054 °C). (b) The observation of $(TiSn)C_x$ and disordered Ti_2SnC in blue frames by HRTEM images from selected areas of 1D-Ti_2SnC at 850 °C reaction with dwell time 0.5 hours.

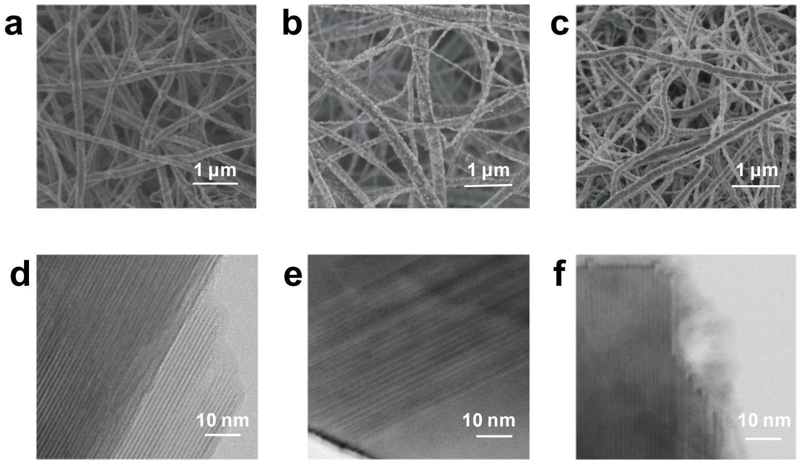

Supplementary Figure 35: SEM images of (a)Ti_3AlC_2, (b) Ti_3SiC_2 and (c) Ti_2SnC nanofiber membranes shown the long-ranged morphologies of obtained 1D-MAX phases. Their corresponding TEM images in (d), (e), and (f) displayed the typical layered structure along $[11\bar{2}0]$ axis.

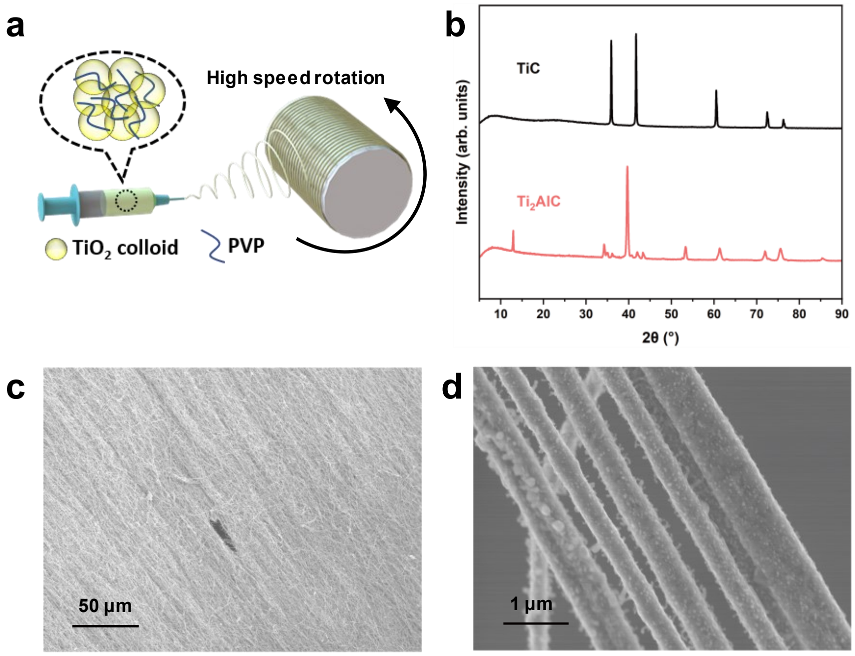

Supplementary Figure 36: Synthesis of aligned TiC and Ti_2AlC nanofibers. (a) Schematic illustration for synthesizing aligned nanofibers by high-speed rotation collectors. (b) XRD patterns of TiC and Ti_2AlC aligned nanofibers. (c) SEM image of aligned nanofibers. (d) Magnified view of aligned nanofibers.

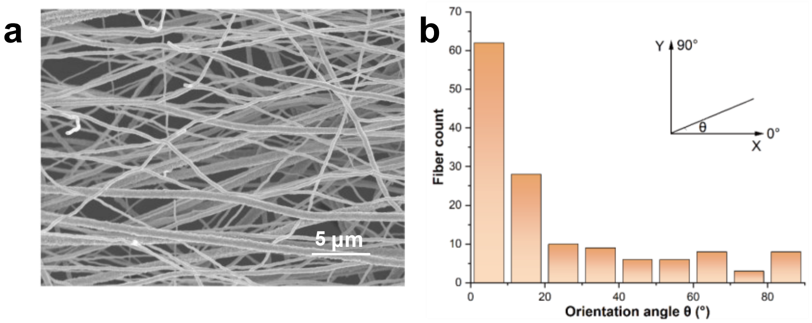

Supplementary Figure 37: Alignment of 1D-Ti_2AlC. (a) SEM image of aligned nanofibers. (b) Statistics by software-processed with the alignments of nanofibers, which showed the most of nanofibers were paralleled at 0°direction.

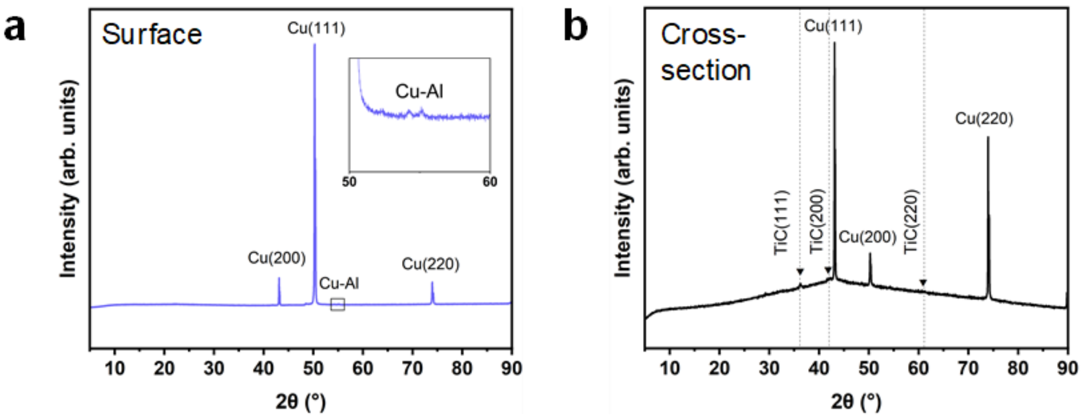

Supplementary Figure 38: XRD patterns of (a) surface, and (b) cross-section of CALF composites.

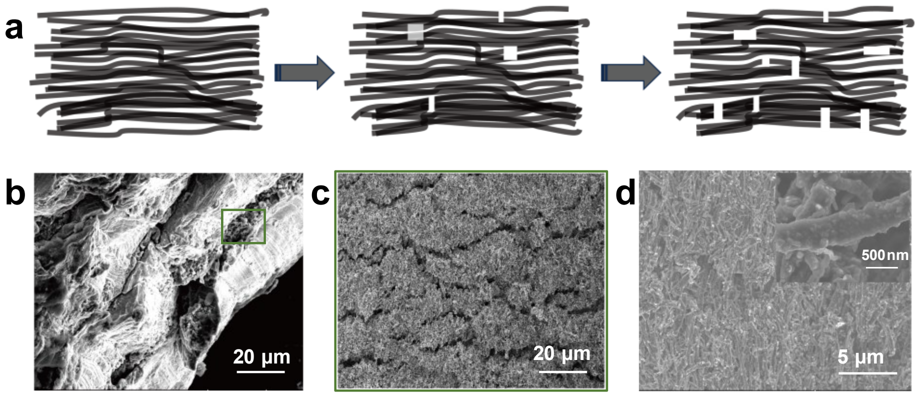

Supplementary Figure 39: Crack motion in aligned nanofibers when applying force on the CALFs. (a) Schematic illustration of crack mechanism with the paralleled loading force. The SEM in figure (b), (c) and (d) shown the crack along one direction, the enlargement in (d) shown the transformation of 1D-Ti_2AlC from nanofibers to nanorods.

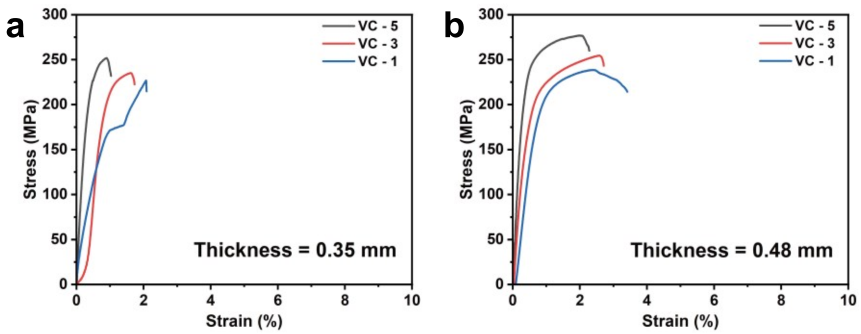

Supplementary Figure 40: Tensile stress−strain curves of CALFs with (a) 0.35 mm, and (b) 0.48 mm thicknesses. The average tensile stresses of thicker CALFs were larger than thinners due to the increased volume contents of reinforced phase ATAC nanofibers.

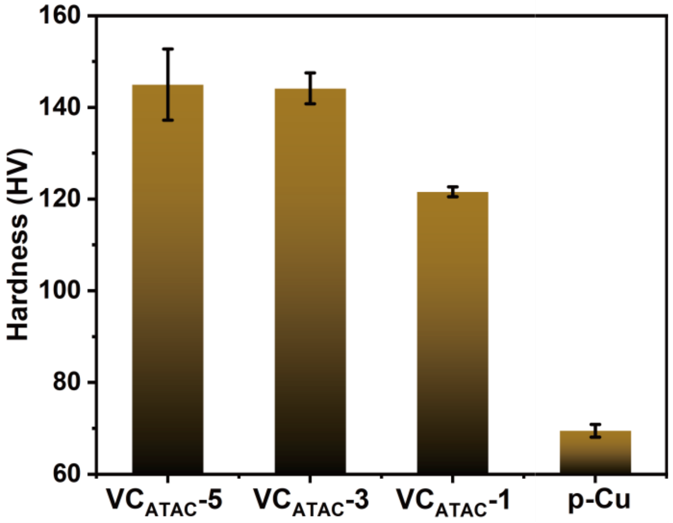

Supplementary Figure 41: The Vickers hardness of CALFs with 0.6 mm average thickness with different volume contents (1%, 3%, and 5%) of reinforcement phase ATAC nanofibers, where the error bars represent the standard error of the mean Vickers hardness for each group, based on n = 3 independent experiments..

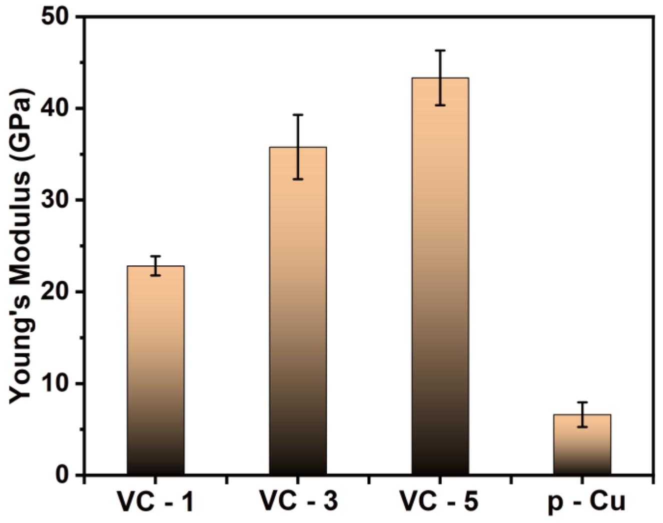

Supplementary Figure 42: Young's modulus of CALFs with 0.6 mm average thickness with different volume contents (1%, 3%, and 5%) of reinforcement phase ATAC nanofibers, where the error bars represent the standard error of the mean Young's modulus for each group, based on n = 3 independent experiments.

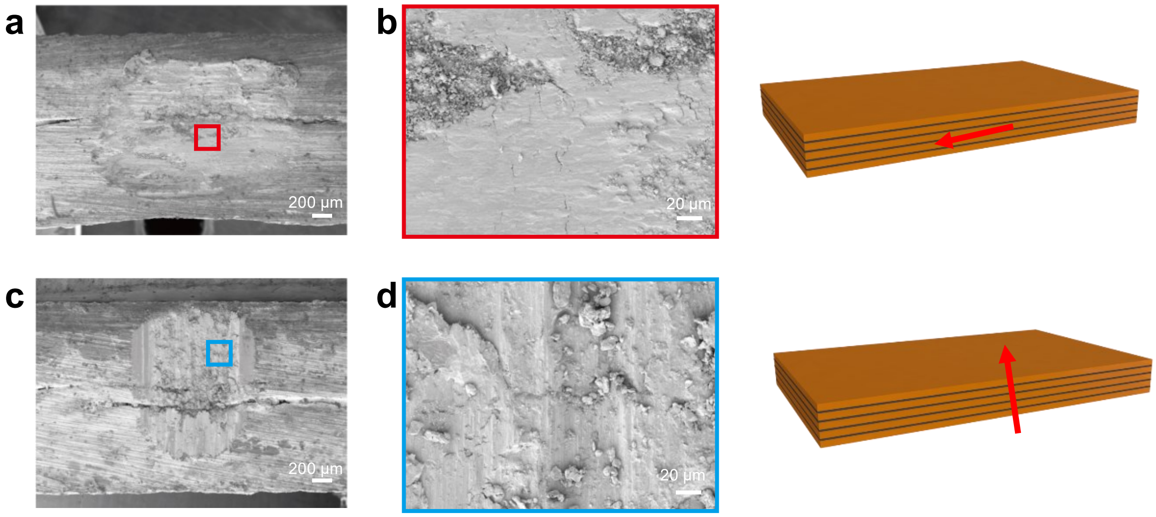

Supplementary Figure 43: SEM micrograph of the cross-section worn surfaces of CALF sliding along different direction, where (a) perpendicular to the stacking direction, (c) parallel to the stacking direction. The enlargements (b) and (d) shows crack, groove and pits, suggesting the plastic deformation in Cu layer to release the friction energy while particle peeling occurred in ceramic layer.

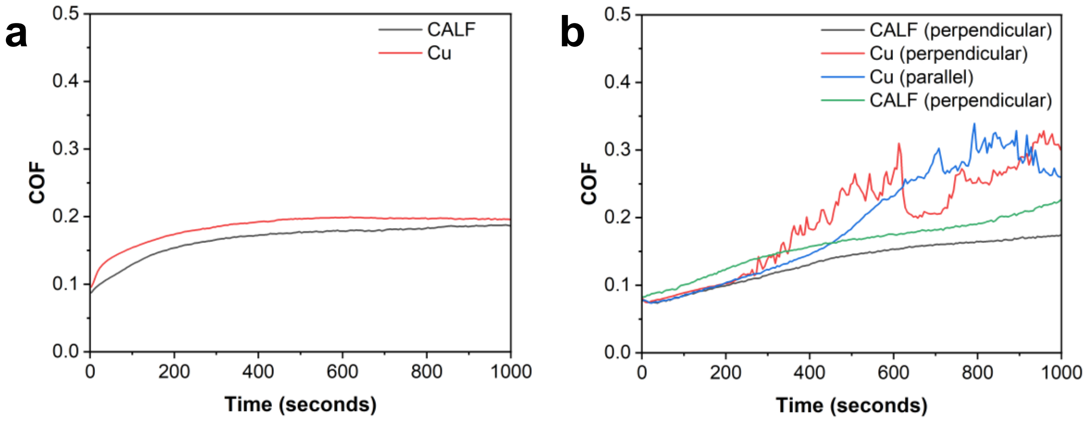

Supplementary Figure 44: Coefficient of friction curves conducted from (a) surface of samples, and (b) cross-section of samples along different directions.

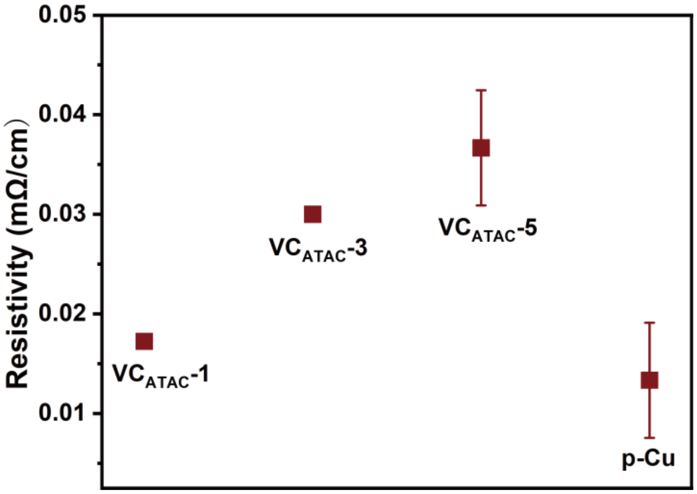

Supplementary Figure 45: Resistivities of CALFs with different volume content (1%, 3%, and 5%) of reinforcement phase ATAC nanofibers, where the error bars represent the standard error of the mean resistivity for each group, based on n = 3 independent experiments..

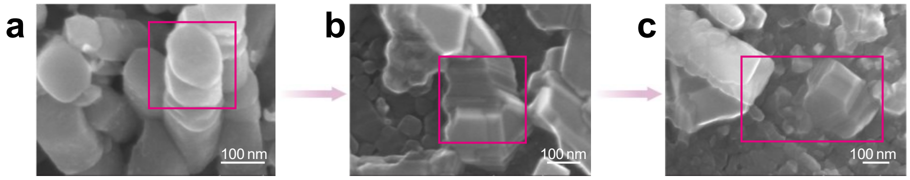

Supplementary Figure 46: SEM images show the HF-etching process in 1D-Ti_2AlC to gradually produce MXenes. At the early stage of etching in (a), the morphologies of 1D-Ti_2AlC turned into slub-shaped, suggesting the MAX phase grains were exposed. With increasing etching time, the layered structure of 1D-MAX phases was emerged in (b). In the final stage, the MAX phases grains peeled off from the nanofibers and the MXene layers were revealed in (c).

Supplementary Table 1.

The reaction conditions of 1D-MAX phases for conformal synthesis.

MAX Phases	Ratio of adding atoms (by mol)	Ratio of atoms and NaCl/KCl eutectic salts (by mol)	T (°C)	Reaction time (hours)
**Ti_2AlC**	Ti: Al = 1:1.2	1: 4	900	2
**Ti_3AlC_2**	Al	1: 1	1000	1
**Ti_3SiC_2**	Ti: Si: Al = 1: 1.2: 0.1	1: 10	1100	1
**Ti_2SnC**	Ti: Sn: Al = 1: 1.2: 0.1	1: 10	850	2

Supplementary Table 2.

Cell parameters and components of prepared 1D-MAX phases obtained by Rietveld analysis.

MAX Phase	Lattice parameter *a* (Å)	Lattice parameter *c* (Å)	wt. %	Reduced χ^2	Rp (%)	GOF	TiC wt. %
**Ti_2AlC**	3.0246	13.6486	95.2	2.83	9.722	1.68	4.8
**Ti_3AlC_2**	3.0583	18.4233	93.7	3.63	11.596	2.15	6.3
**Ti_3SiC_2**	3.0647	17.6866	90.5	3.33	8.592	1.83	9.5
**Ti_2SnC**	3.1508	13.8462	91.4	1.08	5.29	1.04	8.6

Supplementary Table 3.

Comparison of MAX phase products with various micro morphologies from other reports.

Purity (%)	Micro morphology	Bulk morphology	Synthesis temperature (℃)	Method	Ref
＞ 95	Short nanofibers	Powder	900	MS	[13]
＜ 50	Short microfibers	Powder	900	SHS	[14]
＜ 50	Long microfibers	Fiber membrane	900	MS	[15]
＞ 90	Short microrods	Powder	1250	MS	[17]
**＞ 95**	**Long nanofibers**	**Nanofiber membrane**	**900**	**MS**	**This work**

*SHS as in Self-propagating High-temperature Synthesis; MS as in Molten salt synthesis.

Supplementary Table 4.

Parameters obtained for the Ti 2p curve fitting of 1D-Ti_2AlC samples at reaction temperature of 900 °C.

Region	BE (eV)	FWHM (eV)	Fraction	Assigned to
**Ti 2*p*$_{3/2}$(2*p*$_{1/2}$)**	455.04(460.99)	1.10(1.50)	76.98	Ti-C
	458.95 (464.65)	1.47(2.34)	23.02	Ti-O
**C 1*s***	281.3	1.9	6.1	Ti-C
	284.6	2	67	C-C
	285.7	1.9	16.3	C-O
	287.6	3.2	10.6	C=O
**Al 2*p*$_{3/2}$(2*p*$_{1/2}$)**	72.2(72.8)	0.9(0.48)	13.3	Al-Ti
	74.8(75.6)	2.9(1.84)	86.6	Al-O

Supplementary Table 5

The fabrication details of CALFs.

Sample with different thickness (μm)	Amounts of Cu layers	Density (g cm^3)	Volume content of reinforced phase 1D-Ti_2AlC (%)
**CALF (350)**			
T_{Cu}-20	12	8.83	5
T_{Cu}-35	8	8.52	3
T_{Cu}-90	4	8.34	1
**CALF (480)**			
T_{Cu}-20	16	8.76	5
T_{Cu}-35	11	8.43	3
T_{Cu}-90	5	8.11	1
**CALF (600)**			
T_{Cu}-20	20	8.79	5
T_{Cu}-35	14	8.46	3
T_{Cu}-90	6	8.25	1

Note that there were deviations exist in thicknessed of added Ti_2AlC nanofibers membranes, and their surfaces were also not as even as copper foils, therefore the thicknesses recorded in table were measured from three samples each and took the average standards with errors around 10 %, as well as other measured data. The Ti_2AlC nanofibers membranes used had average thicknesses of 10 μm. T_{Cu} refers to the thickness of copper foils.

References and Notes

1 Zhang, F., Si, Y., Yu, J. & Ding, B. Electrospun porous engineered nanofiber materials: A versatile medium for energy and environmental applications. *Chem. Eng. J.* **456**, doi:10.1016/j.cej.2022.140989 (2023).

2 Yu, R., He, L. L. & Ye, H. Q. Effects of Si and Al on twin boundary energy of TiC. *Acta Mater.* **51**, 2477-2484, doi:10.1016/s1359-6454(03)00032-6 (2003).

3 Yang, Q. *et al.* Single-Crystalline Pyramidal TiCx Particles Grown by Biphase Diffusion Synthesis. *ACS Nano* **16**, 7713-7720, doi:10.1021/acsnano.1c11524 (2022).

4 Fang, P. *et al.* Epitaxial TiC (001) layers: Phase formation and physical properties vs C-to-Ti ratio. *Acta Mater.* **226**, doi:10.1016/j.actamat.2022.117643 (2022).

5 Yang, H. *et al.* A new insight into heterogeneous nucleation mechanism of Al by non-stoichiometric TiC_x. *Acta Mater.* **233**, doi:10.1016/j.actamat.2022.117977 (2022).

6 Li, S. *et al.* Advances in Molten Salt Synthesis of Non-oxide Materials. *Energy Environ. Mater.* **6**, doi:10.1002/eem2.12339 (2022).

7 Liu, X., Fechler, N. & Antonietti, M. Salt melt synthesis of ceramics, semiconductors and carbon nanostructures. *Chem. Soc. Rev.* **42**, doi:10.1039/c3cs60159e (2013).

8 Selloni, A., Carnevali, P., Car, R. & Parrinello, M. Localization, hopping, and diffusion of electrons in molten salts. *Phys. Rev. Lett.* **59**, 823-826, doi:10.1103/PhysRevLett.59.823 (1987).

9 Nadimi, H., Soltanieh, M. & Sarpoolaky, H. Molten salt shielded synthesis and formation mechanism of Ti_2AlC in NaCl–KCl medium. *Ceram. Int.* **48**, 9024-9029, doi:10.1016/j.ceramint.2021.12.084 (2022).

10 Zhang, Z. *et al.* Probing the oxidation behavior of Ti_2AlC MAX phase powders between 200 and 1000 °C. *J. Eur. Ceram. Soc.* **37**, 43-51, doi:10.1016/j.jeurceramsoc.2016.08.004 (2017).

11 Magnuson, M. *et al.* Electronic structure and chemical bonding in Ti_2AlC investigated by soft x-ray emission spectroscopy. *Phys. Rev. B.* **74**, doi:10.1103/PhysRevB.74.195108 (2006).

12 Naslund, L.-A., Persson, P. O. A. & Rosen, J. X-ray Photoelectron Spectroscopy of Ti_3AlC_2, $Ti_3C_2T_z$, and TiC Provides Evidence for the Electrostatic Interaction between Laminated Layers in MAX-Phase Materials. *J. Phys. Chem. C.* **124**, 27732-27742, doi:10.1021/acs.jpcc.0c07413 (2020).

13 Barsoum, M. W. & El-Raghy, T. Synthesis and Characterization of a Remarkable Ceramic: Ti_3SiC_2. *J. Am. Ceram. Soc.* **79**, 1953-1956, doi:10.1111/j.1151-2916.1996.tb08018.x (1996).

14 Kang, Y. J., Fey, T. & Greil, P. Synthesis of Ti_2SnC MAX Phase by Mechanical Activation and Melt Infiltration. *Adv. Eng. Mater.* **14**, 85-91, doi:10.1002/adem.201100186 (2012).
